# The Notch Ligand Delta-Like 4 Regulates Multiple Stages of Early Hemato-Vascular Development

**DOI:** 10.1371/journal.pone.0034553

**Published:** 2012-04-13

**Authors:** Ricardo Laranjeiro, Isabel Alcobia, Hélia Neves, Andreia C. Gomes, Pedro Saavedra, Catarina C. Carvalho, António Duarte, António Cidadão, Leonor Parreira

**Affiliations:** 1 Unidade de Biologia da Hematopoiese, Instituto de Histologia e Biologia do Desenvolvimento, Faculdade de Medicina, Universidade de Lisboa, Lisbon, Portugal; 2 Instituto Gulbenkian de Ciência, Oeiras, Portugal; 3 Centro Interdisciplinar de Investigação em Sanidade Animal, Faculdade de Medicina Veterinária, Universidade Técnica de Lisboa, Lisbon, Portugal; Feinberg Cardiovascular Research Institute, Northwestern University, United States of America

## Abstract

**Background:**

In mouse embryos, homozygous or heterozygous deletions of the gene encoding the Notch ligand Dll4 result in early embryonic death due to major defects in endothelial remodeling in the yolk sac and embryo. Considering the close developmental relationship between endothelial and hematopoietic cell lineages, which share a common mesoderm-derived precursor, the hemangioblast, and many key regulatory molecules, we investigated whether Dll4 is also involved in the regulation of early embryonic hematopoiesis.

**Methodology/Principal Findings:**

Using Embryoid Bodies (EBs) derived from embryonic stem cells harboring hetero- or homozygous Dll4 deletions, we observed that EBs from both genotypes exhibit an abnormal endothelial remodeling in the vascular sprouts that arise late during EB differentiation, indicating that this *in vitro* system recapitulates the angiogenic phenotype of Dll4 mutant embryos. However, analysis of EB development at early time points revealed that the absence of Dll4 delays the emergence of mesoderm and severely reduces the number of blast-colony forming cells (BL-CFCs), the *in vitro* counterpart of the hemangioblast, and of endothelial cells. Analysis of colony forming units (CFU) in EBs and yolk sacs from Dll4^+/−^ and Dll4^−/−^ embryos, showed that primitive erythropoiesis is specifically affected by Dll4 insufficiency. In Dll4 mutant EBs, smooth muscle cells (SMCs) were seemingly unaffected and cardiomyocyte differentiation was increased, indicating that SMC specification is Dll4-independent while a normal dose of this Notch ligand is essential for the quantitative regulation of cardiomyogenesis.

**Conclusions/Significance:**

This study highlights a previously unnoticed role for Dll4 in the quantitative regulation of early hemato-vascular precursors, further indicating that it is also involved on the timely emergence of mesoderm in early embryogenesis.

## Introduction

The first hematopoietic cells in the mouse embryo appear shortly after gastrulation, around embryonic day (E) 7.5, in the blood islands of the yolk sac (YS), in close association with endothelial cells, corresponding to a transient population of nucleated primitive erythrocytes. Primitive erythropoiesis is followed, between E8.25 and E10.5, by a second YS hematopoietic wave in which definitive erythro-myeloid progenitors are generated [1]. *In vitro* and *in vivo* studies strongly suggest that all YS hematopoietic cells derive from the hemangioblast, a common precursor of hematopoietic and endothelial cell lineages [2]–[4]. Subsequently, the fetal liver, first colonized by YS-derived hematopoietic progenitors and then by hematopoietic stem cells (HSCs) arising in the intraembryonic aorta-gonad-mesonephros (AGM) region, becomes the primary hematopoietic organ during fetal development. Toward the end of gestation, HSCs migrate to the bone marrow, where hematopoiesis is maintained throughout post-natal life [1], [5].

One of the major signaling pathways known to regulate several hematopoietic developmental stages and microenvironments is the Notch pathway [6]. In mammals, five Notch ligands, of the Delta-like (Dll1, Dll3 and Dll4) and Jagged (Jag1 and Jag2) families can interact with four Notch receptors (Notch1-4) leading to sequential proteolytic cleavages of the receptor that release the Notch intracellular domain (NICD) from the plasma membrane. The NICD migrates to the nucleus where it binds to the transcription factor RBP-Jk, inducing the expression of several target genes by the recruitment of co-activators [6].

The role of Notch signaling in post-natal hematopoiesis has been extensively studied [7] but its function in embryonic hematopoiesis, especially in the YS stage, is much less understood. Notch1 is required for the generation of long-term HSCs at the AGM, yet it appears to be dispensable for YS hematopoiesis [8], [9]. However, several reports show that Notch signaling plays a role in mesodermal differentiation and specification into the respective cell lineages, which include cardiac muscle, mural, endothelial and hematopoietic cells [9]–[13]. In fact, Notch1 signaling is active during mouse gastrulation in nascent mesoderm and YS blood islands [14], suggesting that it might have a physiological role in early stages of mesoderm commitment and, particularly, in the generation of early hematopoietic precursors. However, which specific Notch ligands are involved in Notch activation during YS hematopoiesis is completely unknown. The Notch ligand Dll4 is, in this respect, of particular interest. Mouse embryos carrying homozygous or heterozygous deletions of the Dll4 gene are grossly similar to their normal counterparts until E8.75–9.0 when a developmental delay becomes apparent. This is followed by death of all Dll4^−/−^ and (the majority) of Dll4^+/−^ embryos at E9.5 and E10.5, respectively, due to severe angiogenic defects in the YS and embryo [15]–[17]. The close developmental relationship between endothelial and hematopoietic cell lineages together with the fact that endothelium itself is an essential microenvironment for the generation of hematopoietic precursors [18]–[20], makes it plausible that Dll4 might also be involved in the regulation of YS hematopoiesis.

To address this issue we have made use of mouse embryonic stem (ES) cell-derived EBs, an *in vitro* model that faithfully recapitulates the *in vivo* dynamics of YS hematopoiesis [21], [22]. Analysis of EBs generated from Dll4^+/−^ and Dll4^−/−^ ES cells show that Dll4 regulates the timely emergence of mesoderm during EB differentiation and its potential to form BL-CFCs, the precursors of endothelial and hematopoietic cells in this experimental system [3]. We also observed that a normal dose of Dll4 is required for the emergence of primitive erythropoiesis (both *in vitro* and *in vivo*), for the correct remodeling of endothelial cells and for the generation of normal numbers of cardiomyocytes and endothelial cells, while the specification of SMCs is Dll4-independent.

## Results

### Expression of Dll4 during EB differentiation

**Figure 1:**
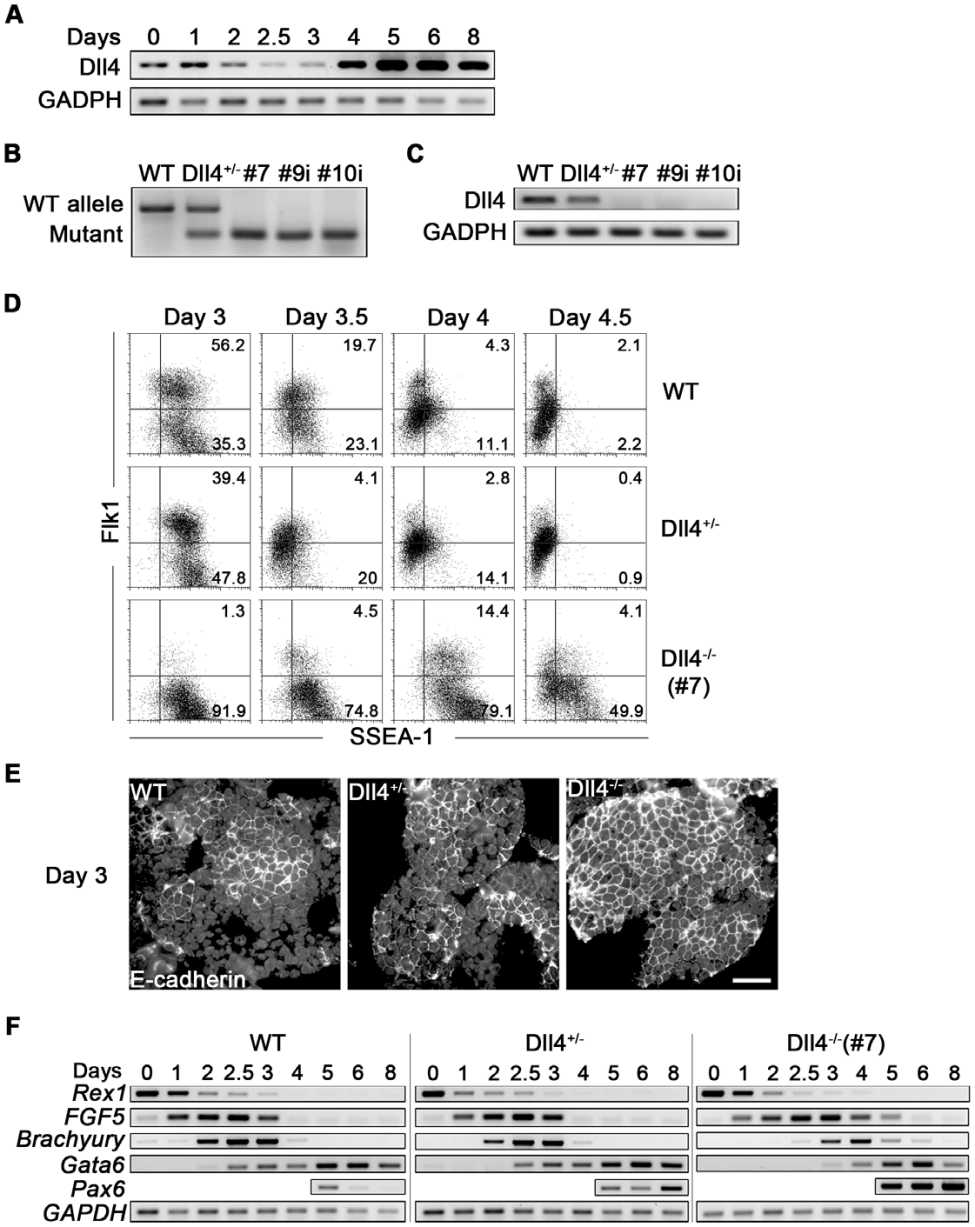
Generation and characterization of Dll4 mutant EBs. (A) Semi-quantitative RT-PCR analysis of *Dll4* expression in EBs generated from WT-R1 ES cells, from day 0 to day 8. Data representative of three independent experiments. (B) PCR genotyping of WT and mutant Dll4 alleles of WT, Dll4^+/−^ and three Dll4^−/−^ (#7, #9i and #10i) ES cell lines. (C) Semi-quantitative RT-PCR analysis of *Dll4* expression in WT, Dll4^+/−^ and Dll4^−/−^ EBs with 3 days of differentiation. (D) Representative flow cytometry dot-plot of SSEA-1/Flk1 expression in WT, Dll4^+/−^ and Dll4^−/−^ EBs from day 3 to day 4.5. (E) Immunofluorescence detection of E-cadherin (membrane bright staining), counterstained with DAPI (nuclear gray staining), of WT, Dll4^+/−^ and Dll4^−/−^ (#7) EBs with 3 days of differentiation. (F) Semi-quantitative RT-PCR analysis of *Rex1*, and genes involved in the formation of germ layers in WT, Dll4^+/−^ and Dll4^−/−^ EBs from day 0 to day 8 of differentiation. Data representative of three independent experiments. Scale bar represents 10 µm.

Analysis of Dll4 expression in EBs differentiated from the parental R1 (WT) ES cell line by semi-quantitative RT-PCR showed that Dll4 is already expressed in undifferentiated ES cells, becomes transiently up-regulated at day 1, down-regulated from days 2 to 3, and then strongly expressed from day 4 until day 8 (Figure 1A). This expression dynamics is compatible with a role for Dll4 in the EB pre-hemangioblast stage [23] and, subsequently, at the time window at which primitive and definitive hematopoietic progenitors are generated [21].

### Effects of Dll4 in ES cell maintenance and EB generation

To address the role for Dll4 in embryonic hematopoiesis we used a genetically modified R1 ES cell line in which one Dll4 allele has been disrupted by insertion of a *lacZ-neo* cassette (Dll4^+/−^ ES cells) [15]. Dll4^−/−^ clones were obtained from these cells by G418 selection. PCR genotyping of G418 resistant colonies revealed that the WT Dll4 allele had been lost in three ES cell clones (#7, #9i and #10i; Figure 1B) where the absence of Dll4 mRNA was confirmed by RT-PCR (Figure 1C).

Dll4^+/−^ and Dll4^−/−^ ES cell colonies exhibited normal undifferentiated morphology (not shown) and similar expansion rate when compared to WT ES cells (Figure S1A). When allowed to form EBs in liquid culture, no significant differences were observed among WT and Dll4 mutant ES cell lines as to number of generated EBs or EB-cellularity, cell-proliferation or apoptosis (Figure S1 B–E). However, when the expression of a pluripotentiality marker, stage-specific embryonic antigen-1 (SSEA-1) [24], was analysed throughout EB development (percentage and absolute numbers are in Figure S1F and Figure S2A, respectively), a more sustained SSEA-1 expression was observed in Dll4^−/−^ EBs from day 3 until day 6, when compared to WT and Dll4^+/−^ EBs. This finding suggests that Dll4 influences ES cell differentiation. Co-expression of SSEA-1 and Flk1, which is representative of early mesodermal progenitors [25], is in fact patent in day 3 WT and Dll4^+/−^ EBs but only becomes evident at day 4 in Dll4^−/−^ EBs (Figure 1D; absolute numbers in Figure S2B). This observation suggests a delay in the differentiation pathway towards mesoderm lineages in Dll4 null EBs. Also, the immuno-detection of E-cadherin in 3D preserved EBs showed that Dll4^−/−^ EBs maintain an epiblast-like morphology at day 3, whereas in both WT and Dll4^+/−^ EBs a mesoderm-like cell population (loosely connected E-cadherin^−^ cells) is clearly visible surrounding a compartment of E-cadherin^+^ cells at the same time point (Figure 1E).

### Dll4 regulates the emergence of germ layers during EB development

The above results assign a regulatory action of Dll4 in the timely emergence of mesodermal cells. To further clarify this issue, we analysed the expression pattern of several genes known to mark the formation of mesoderm, and the other germ layers, during EB differentiation. As shown in Figure 1F, the down-regulation of the pluripotentiality marker *Rex1* [26] and the emergence of the primitive ectoderm marker *FGF5* [27] was not apparently affected in Dll4 mutant EBs. However, in Dll4^−/−^ EBs, *FGF5* expression was maintained until day 5, while in WT and Dll4^+/−^ EBs it was only present until day 3 of differentiation, suggesting a delay in the differentiation of ES cells when Dll4 is absent. Consistent with this notion, the appearance of the mesodermal marker *Brachyury* [28] was 24 hours delayed (and its levels reduced) in Dll4^−/−^ EBs (Figure 1F and Figure S1G). The emergence of primitive endoderm, as assessed by *Gata6* expression [29], was also delayed in Dll4^−/−^ EBs. In contrast, increased expression of the neuroectodermal gene *Pax6* [30] was observed in Dll4^+/−^ and in Dll4^−/−^ EBs (more pronounced in the latter; Figure 1F).

These results show that Dll4 is not essential for ES cell maintenance while strongly suggesting that it regulates the timing of germ layer emergence during EB development, promoting mesoderm formation while inhibiting that of neuroectoderm.

### Dll4 regulates the generation of Flk1^+^ mesodermal cells and hemangioblasts

**Figure 2:**
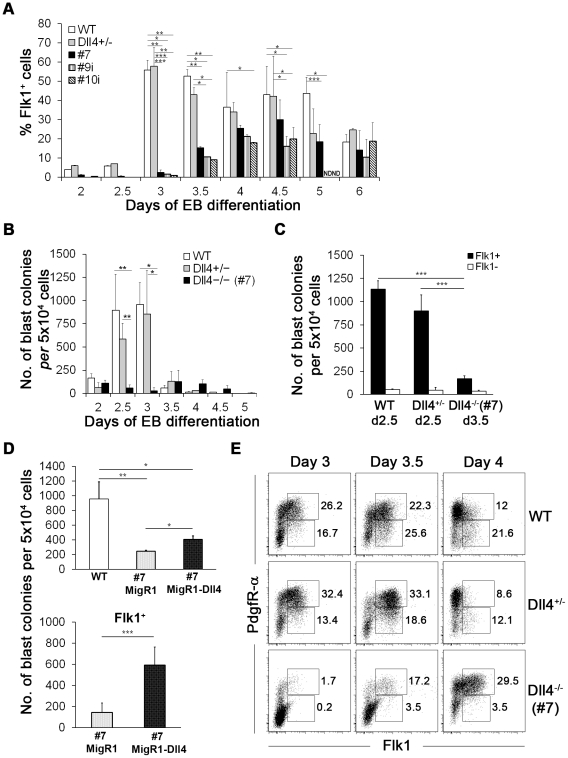
Analysis of Flk1 expression and BL-CFC potential of WT and Dll4 mutant EBs. (A) Flow cytometry analysis of Flk1 expression in WT, Dll4^+/−^ and Dll4^−/−^ EBs from day 2 to day 6 of differentiation. (B) Number of blast colonies generated by WT, Dll4^+/−^ and Dll4^−/−^ EB cells from day 2 to day 5 of differentiation. (C) Number of blast colonies generated by sorted Flk1 cells derived from day 2.5 WT and Dll4^+/−^ EBs and from day 3.5 Dll4^−/−^ EBs. (D) Number of blast colonies generated by day 3 EBs (upper) or sorted Flk1 cells (bottom) differentiated from WT and Dll4^−/−^ ES cells transduced with empty (pMigR1) or recombinant virus (pMigR1-Dll4). (E) Representative flow cytometry dot-plot of Flk1/PdgfR-α expression from day 3 to day 4 of WT, Dll4^+/−^ and Dll4^−/−^ EBs (Flk1^+^/PdgfR-α^+^: day 3 - WT and Dll4^+/−^ *versus* #7, 0.00060<*P*<0.0406; Flk1^+^/PdgfR-α^−^: day 3 to day 4 - WT and Dll4^+/−^ *versus* #7, 0.0002<*P*< 0.0094; day 4 - WT *versus* Dll4^+/−^, *P* = 0.0457). ND not determined. *P<.05, **P<.01, ***P<.001.

The earliest marker of mesodermal differentiation towards hemato-vascular development is Flk1, the receptor-2 of vascular endothelial growth factor (VEGF) [31], [32]. As shown in Figure 2A, WT and Dll4^+/−^ EB cells exhibited similar dynamics of Flk1 expression, with the first Flk1^+^ cells appearing at day 2, followed by a peak 24 hours later when near 50% of EB cells become Flk1^+^. In contrast, Dll4^−/−^ EBs showed a 24 hour delay in the emergence of this cell population, being almost devoid of Flk1^+^ cells until day 3 of differentiation. Thereafter, Flk1^+^ cells increased, reaching its peak (not exceeding 30%) at day 4 (Figure 2A; absolute cell numbers in Figure S2C). Flk1 expression dynamics is in fact similar to that of cells co-expressing SSEA-1 and Flk1 (see above; Figure S2B), which represent early mesodermal progenitors [25]. Thus, the absence of Dll4 delays the emergence of Flk1^+^ cells and severely reduces their number in differentiating EBs.

As hemangioblasts are included in the earliest Flk1^+^ subpopulation [23], we proceeded to analyze the potential of WT, Dll4^+/−^ and Dll4^−/−^ EBs to generate BL-CFCs, from day 2 to day 5. As shown in Figure 2B, WT EBs exhibited the characteristic transient wave of BL-CFC potential [2], with cells from day 2.5 and day 3 EBs generating the highest number of blast colonies. Similar kinetics was observed in Dll4^+/−^ EBs, which produced a reduced (but statistically non-significant) number of blast colonies, at days 2.5 and 3, when compared to those of control EBs. A striking reduction in the number of blast colonies was, however, observed in Dll4^−/−^ EBs at these time-points. Similar results were obtained in EBs derived from the other Dll4 null clones (analysed at day 3; Figure S3A). The capacity to generate BL-CFC was further assessed in sorted Flk1^+^ *versus* the Flk1^−^ EB cell population. The analysis was performed at day 2.5 for WT and Dll4^+/−^ EBs, and at day 3.5 for Dll4^−/−^ EBs, because the emergence of Flk1^+^ mesoderm was found to be delayed 24 hours in the absence of Dll4 (see above). In all cases, the BL-CFC potential was virtually absent from Flk1^−^ EB cells, and a significantly decreased capacity to generate BL-CFC was observed in Flk1^+^ cells sorted from Dll4^−/−^ EBs when compared to those isolated from WT and Dll4^+/−^ EBs (Figure 2C). A close inspection throughout the sequential stages of blast colony development [33] revealed that Dll4 null (and Dll4^+/−^) blast colonies were always morphologically undistinguishable from those generated by WT cells (not shown). These results strongly suggest that the absence of Dll4 severely decreases the emergence of Flk1^+^ cells in the EB and the ability of these cells to differentiate into BL-CFC.

To further confirm that the BL-CFC phenotype of Dll4^−/−^ EBs was due to loss of Dll4, Dll4^−/−^ ES cells (#7) were transduced with a retrovirus containing the full-length Dll4 cDNA and blast colony assays performed at day 3 of EB differentiation from total and Flk1^+^ sorted cells. At this time point, only 30–50% of EB cells expressed GFP, an indication that retroviral silencing has occurred in the remaining cells [34]. Despite this fact, Dll4-transduced EBs still generated significantly higher numbers of blast colonies when compared to those transduced with the empty virus (control; Figure 2D). The percentage of Flk1^+^ EB cells, which was found to be reduced in Dll4^−/−^ EBs (see above), also significantly increased in Dll4-transduced day 3 EBs when compared to the control (Figure S3B).

The hematopoietic subpopulation of Flk1^+^ cells can be distinguished from Flk1^+^ cells exhibiting cardiac potential by the co-expression of PdgfR-α in the latter [35]. As shown in Figure 2E the vast majority of Flk1^+^ cells from Dll4^−/−^ EBs co-expressed PdgfR-α, indicative of its enhanced cardiac differentiation and reduced hemangiogenic potential when compared with WT and Dll4^+/−^ Flk1^+^ cells (see below).

Together, these results show that the absence of Dll4 delays the emergence of Flk1^+^ mesodermal cells in the EB and, within this population, reduces the generation of cells with hemangioblast potential.

### Dll4 heterozygosity specifically affects primitive erythropoiesis both *in vitro* and *in vivo*

**Figure 3:**
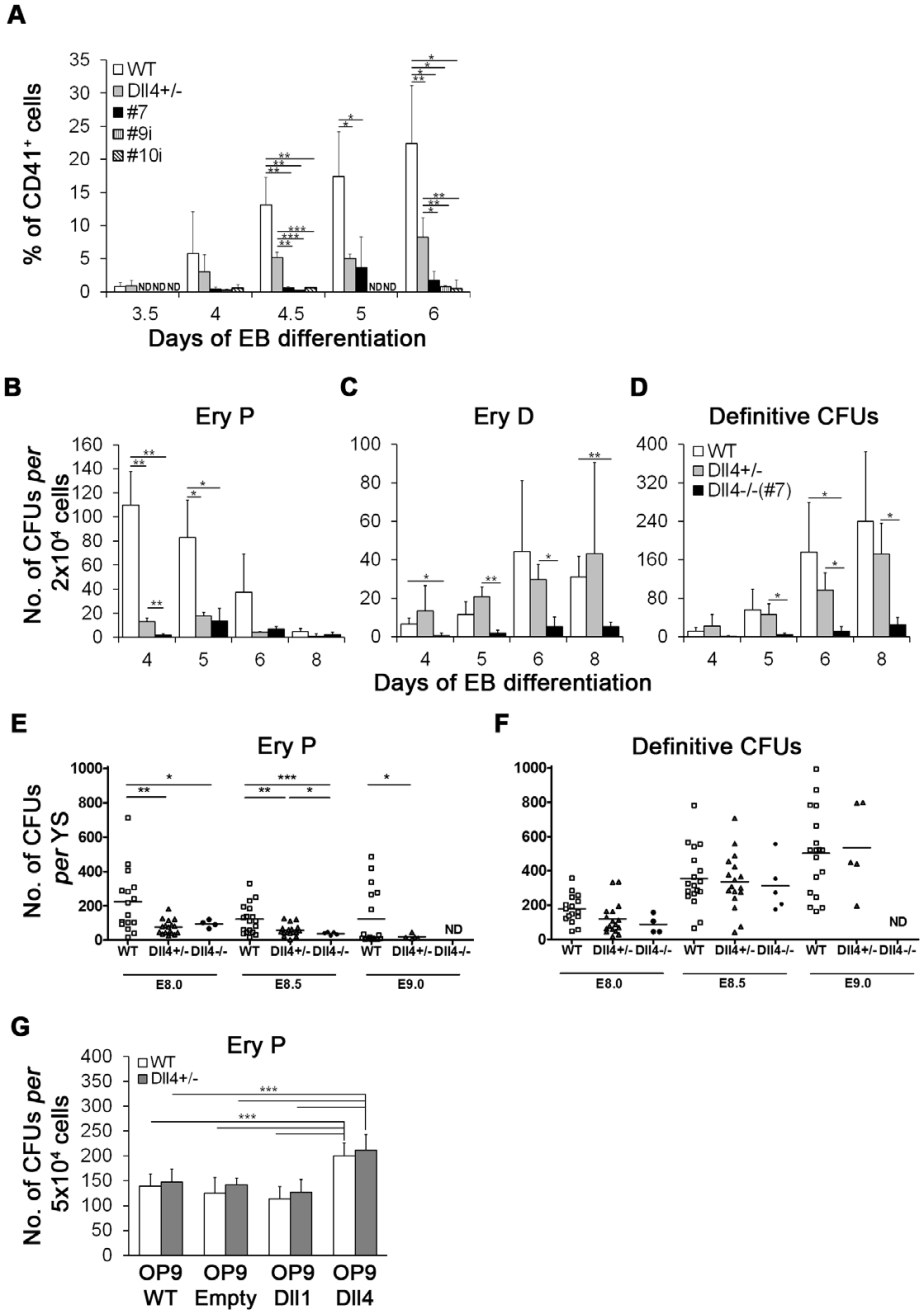
Effects of Dll4 in hematopoiesis. (A) Flow cytometry analysis of CD41 expression in WT, Dll4^+/−^ and Dll4^−/−^ EBs from day 3.5 to day 6 of differentiation. (B) Number of CFU-EryP; (C) CFU-definitive erythroid (EryD) and (D) total definitive CFUs (include CFU-EryD, -macrophage, -granulocyte, -granulocyte//macrophage and -erythroid/granulocyte/macrophage) generated by WT, Dll4^+/−^ and Dll4^−/−^ EB cells at days 4, 5, 6 and 8 of differentiation. (E–F) Number of CFU-EryP (E) and total definitive CFUs (F) generated by YS cells from WT, Dll4^+/−^ and Dll4^−/−^ mouse embryos at E8.0, E8.5 and E9.0. Each symbol represents individual YS and horizontal lines indicate the mean value. (G) Number of CFU-EryP generated by WT and Dll4^+/−^ cells at day 5 of differentiation on parental OP9 cells (OP9-WT) or OP9 cells transduced either with retroviral vectors containing Dll4 (OP9-Dll4), Dll1 (OP9-Dll1) or only IRES-eGFP sequences (OP9-Empty). ND not determined. *P<.05, **P<.01, ***P<.001.

We then investigated the effects of mono- and bi-allelic deletion of Dll4 on the generation of hematopoietic cells. The expression of CD41, the earliest marker of embryonic hematopoietic progenitors [36] was analysed during EB differentiation. When compared to WT EBs, a clear reduction in CD41^+^ cells was observed in Dll4 mutant EBs, more pronounced in Dll4^−/−^ EBs (Figure 3A; absolute numbers in Figure S2D). Consistent with these observations, decreased expression of *Scl*, *Gata1*, and *Runx1*, transcription factors known to be essential for hemangioblast/hematopoietic development [22], was observed in Dll4^−/−^ EBs (Figure S4A).

While these results indicate that Dll4 mutant EBs have reduced hematopoietic potential, they do not discriminate between a generalized hematopoietic problem and a restricted cell-lineage defect. The capacity of EB cells to generate specific colony forming units (CFUs) was thus investigated, from day 4 to day 8 of EB differentiation. Dll4^+/−^ EBs showed a dramatic reduction in the number of primitive erythroid (EryP) progenitors relative to control EBs (Figure 3B). However, definitive hematopoietic progenitors were not significantly reduced in Dll4^+/−^ EBs, indicating that definitive erythro-myeloid progenitors remain almost unaffected by Dll4 heterozygosity (Figure 3C–D). In contrast, Dll4^−/−^ EBs exhibited a strong reduction in all hematopoietic progenitors (Figure 3B–D), consistent with their diminished hemangioblast potential. Once again, no morphological differences were observed in Dll4 mutant colonies when compared with the control (not shown).

To determine if YSs derived from Dll4 mutant embryos have a similar hematopoietic phenotype to that observed *in vitro*, CFU analysis was also performed in YSs derived from WT, Dll4^+/−^ and Dll4^−/−^ mouse embryos. As shown in Figure 3E, a severe reduction in the number of EryP progenitors was observed in both Dll4^+/−^ and Dll4^−/−^ YSs at E8.0, E8.5 and E9.0 (Dll4^−/−^ YSs not analyzed at E9.0) when compared to the control. However, definitive erythro-myeloid progenitors were not reduced in Dll4 mutant YSs, indicating that Dll4 is not essential for the generation of definitive YS hematopoietic progenitors *in vivo* (Figure 3F).

To better define the role of Dll4 in primitive erythropoiesis, namely to probe for a Dll4-specific positive influence, WT and Dll4^+/−^ ES cells were differentiated on parental OP9 cells (OP9-WT) or OP9 cells transduced either with retroviral vectors containing Dll4 (OP9-Dll4), Dll1 (OP9-Dll1) or only IRES-eGFP sequences (OP9-Empty) [37], and then further plated for primitive erythroid colony assays. As shown in Figure 3G, WT and Dll4^+/−^ cells differentiated on OP9-Dll4 generated a higher number of primitive erythroid colonies when compared to the ones from control feeder layers (OP9-WT and OP9-Empty), or to those co-cultured on OP9-Dll1 cells. The lower EryP potential of the Dll4^+/−^ cells found in the EB differentiation system (see above) did not occur in OP9 co-cultures. While no further experiments were performed to specifically address this issue, it is plausible that such different behaviour may be related to the fact that, in Dll4^+/−^ EBs, all potentially signalling-cells exhibit Dll4 heterozygosity, while in OP9 co-cultures Dll4^+/−^ ES cells were differentiated on feeder layers expressing normal (e.g. OP9-WT) or higher Dll4 levels (OP9-Dll4).

Our data shows that the notch-ligand Dll4 differentially influences the generation and development of specific hematopoietic cell lineages. Importantly, the lack of one Dll4 allele was sufficient to induce a marked reduction of primitive erythropoiesis, both *in vitro* and *in vivo*. Additionally, our results from OP9 co-cultures showed that exposure to higher levels of Dll4, but not of Dll1, results in increased primitive erythropoiesis, stressing the hypothesis of a ligand-specific effect.

### Dll4 mutant EBs display endothelial remodelling defects

**Figure 4:**
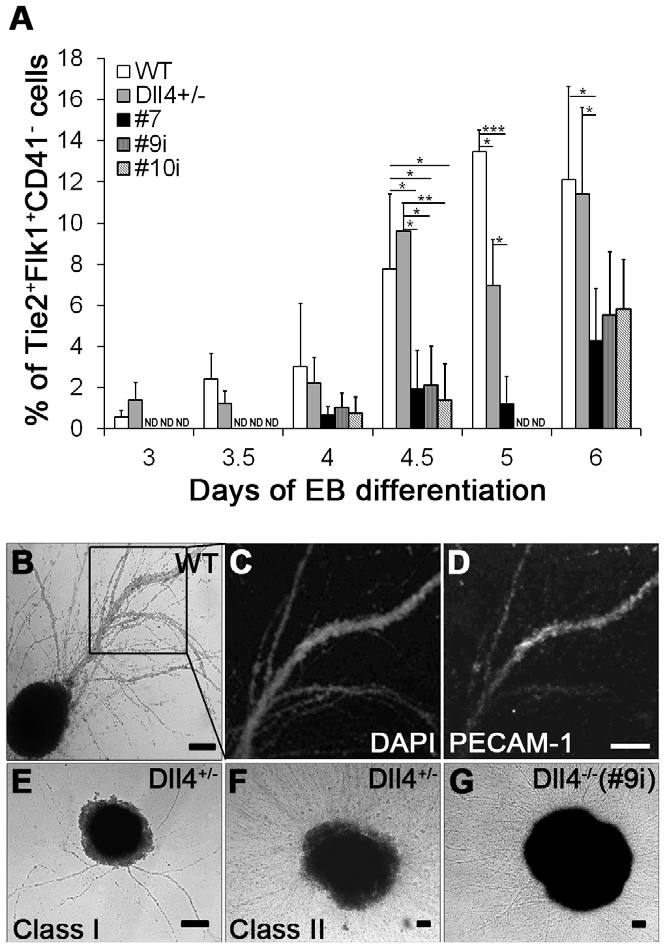
Endothelial differentiation and remodelling defects in Dll4 mutant EBs. (A) Flow cytometry analysis of Tie2^+^Flk1^+^CD41^−^ cells in WT, Dll4^+/−^ and Dll4^−/−^ EBs from day 3 to day 6 of differentiation. (B) Bright field image of a WT EB, at day 25 of differentiation, showing sprouts in methylcellulose medium. (C–D) Inset shows the correspondent confocal images for DAPI (C) and PECAM-1 (D) staining of EB sprouts. (E–G) Representative bright field images of Dll4^+/−^ (class I and II sprouts; E–F) and Dll4^−/−^ (clone #9i; G) EBs with endothelial sprouts, cultured for 25 days in methylcellulose medium. Scale bar represents 100 µm. ND not determined. *P<.05, **P<.01, ***P<.001.

The endothelium is another cell lineage derived from the hemangioblast and, as well, from a subpopulation of Flk1^+^ cells known to appear at day 4.25 of EB development [38]. Therefore, endothelial differentiation was investigated throughout EB development. The percentage and number (Figure 4A, and Figure S2E, respectively) of endothelial cells (Tie2^+^/Flk1^+^/CD41^−^ cells) in Dll4^+/−^ EBs was found to be similar to those of control EBs, with the exception of day 5 where a significant reduction in the percentage of these cells was seen. A severe reduction of endothelial cells was consistently present in Dll4^−/−^ EBs, once again in agreement with the reduction of their hemangioblast potential and Flk1 expression.

At later stages of differentiation (beyond day 10), EBs cultured in methylcellulose medium developed sprouts (Figure 4B–G) which were positive for the endothelial cell marker CD31 (PECAM-1) (Figure 4D). Striking morphological differences were found between WT and Dll4 mutant EBs. As shown in Figure 4B–D, in the former, sprouts were well defined, frequently branched and made of two parallel cell monolayers separated by a lumen. In contrast, Dll4^+/−^ EB sprouts were generally formed by one single monolayer of cells without a visible lumen and with less frequent branching. Moreover, two classes of Dll4^+/−^ EBs were found, both maintaining the described endothelial sprout morphology but differing in sprout quantity: with very few sprouts (class I) or with abundant sprouts (class II) (Figure 4E and F). Dll4^−/−^ EBs with endothelial sprouts exhibited a phenotype very similar to class II Dll4^+/−^ EBs (Figure 4G).

Together, our data show that Dll4 mutant EBs are capable of generating endothelial cells (albeit at reduced levels in Dll4^−/−^ EBs) but the remodelling of these cells into normal vascular structures is severely affected, akin to what happens in Dll4 mutant embryos.

### Dll4 is not essential for smooth muscle cell differentiation but inhibits cardiomyogenesis

**Figure 5:**
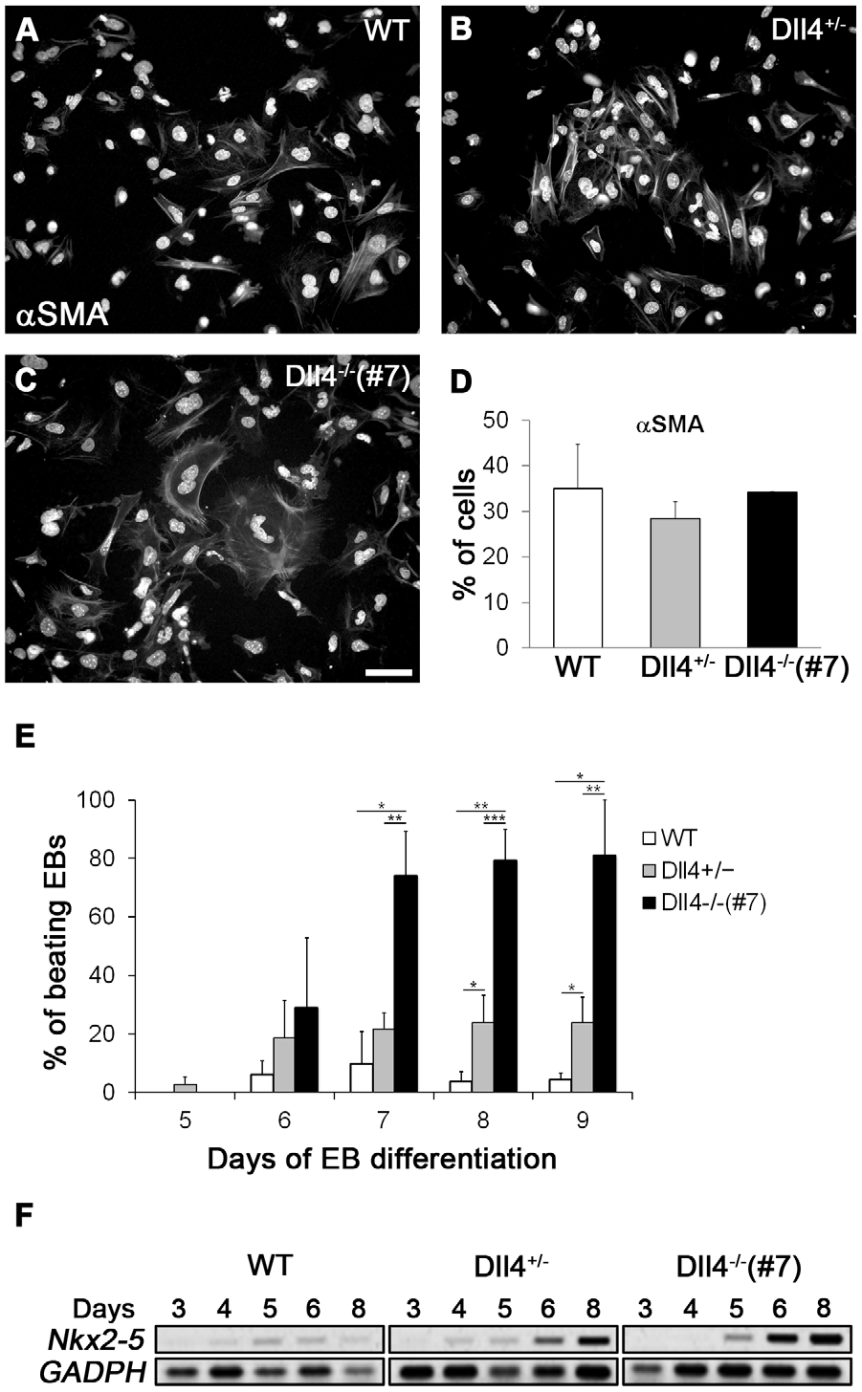
Differential effects of Dll4 on the emergence of SMCs and cardiomyocytes. (A–C) Representative immunofluorescence images for αSMA (cytoplasmic positive fibers), counterstained with DAPI (nuclear staining), of day 3 EB-derived cells cultured for 5 days in monolayer culture. (D) Percentage of αSMA^+^ cells obtained from day 3 EB-derived cells of WT, Dll4^+/−^ and Dll4^−/−^ cell lines. (E) Percentage of beating EBs in suspension culture from day 5 to day 9 of differentiation. (F) Semi-quantitative RT-PCR analysis of *Nkx2-5* in WT, Dll4^+/−^ and Dll4^−/−^ EBs from day 3 to day 8 of differentiation. Scale bar represents 10 µm. *P<.05, **P<.01, ***P<.001.

As SMCs are important for vascular maturation and remodelling, they might play a role in the angiogenic defects observed in Dll4 mutant EBs. To investigate the effects of Dll4 on smooth muscle differentiation, cells isolated from day 3 EBs were cultured in matrigel-coated wells for 5 days in the presence of VEGF and basic fibroblast growth factor (bFGF), conditions known to promote the proliferation and differentiation of SMCs and endothelial cells [39]. The presence of SMCs was then assessed by immuno-detection of α-smooth muscle actin (αSMA) (Figure 5A–C). Similar proportions of SMCs were observed in WT, Dll4^+/−^ and Dll4^−/−^ cultures (Figure 5D). In contrast, endothelial cells (as determined by flow cytometry analysis of Tie2^+^/Flk1^+^/VE-cadherin^+^ cells; not shown) were markedly reduced in Dll4^−/−^ cultures when compared to their WT and Dll4^+/−^ counterparts (0.4%±0.2 *versus* 20.74%±9.7 in WT and, 13.6%±3.8 in Dll4^+/−^ conditions; *P*<0.05), confirming that the absence of Dll4 specifically hampers the emergence of this cell lineage (see data from EB cultures in Figure 4A).

As BL-CFCs also have the potential to generate vascular SMCs [40], blast colonies with 4 days of development were also cultured as described above [39]. After 4 days, SMCs were present at similar levels in cultures of WT, Dll4^+/−^ and Dll4^−/−^ blast colony-derived cells (not shown). These results indicate that differentiation of SMCs either from the hemangioblast or from other EB cells with SMC potential [38] is not affected by diminished Dll4 signalling.

The generation of cardiomyocytes, another mesoderm-derived cell lineage generated in developing EBs [35], [38], [41] was analyzed by the direct visualization of rhythmic contractile regions that spontaneously appear during EB differentiation and, by expression of the *Nkx2-5* gene, one of the earliest cardiac marker genes [42]. As shown in Figure 5E, the proportion of EBs with contractile regions (beating EBs) was significantly higher in Dll4^+/−^ and Dll4^−/−^ EBs than in WT EBs, with most of the latter EBs exhibiting contractile regions beyond day 7 of differentiation. This observation is supported by our finding that the vast majority of Flk1^+^ cells from Dll4 null EBs co-express PdgfR-α, indicative of enhanced cardiac differentiation [35], when compared with the WT and Dll4^+/−^ Flk1^+^ cell population (see above; Figure 2E). Accordingly, an increase in *Nkx2-5* expression was observed in Dll4^+/−^ and Dll4^−/−^ EBs, when compared with WT EBs (Figure 5F).

Together, our results indicate that Dll4 affects the differentiation of distinct mesoderm-derived cell lineages in different ways, promoting hemato-vascular potential while inhibiting cardiomyogenesis.

## Discussion

**Figure 6:**
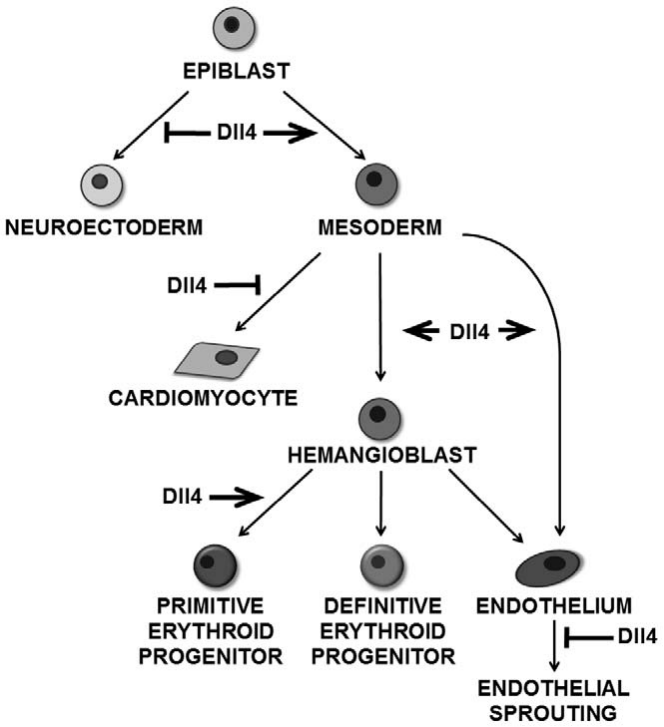
Model for Dll4 influence on ES cell differentiation. Model depicting the role of Dll4 at multiple stages of ES cell differentiation.

We showed that Dll4 is involved in the timely emergence of mesoderm in differentiating EBs, subsequently favoring the generation of hemangioblasts and primitive erythroid cells. We also observed that Dll4 has differential effects in other mesoderm-derived cell lineages, promoting endothelial differentiation, inhibiting cardiomyogenesis while not affecting the specification of SMCs (Figure 6).

Hemi or homozygous deletions of Dll4 did not affect the morphology and growth of undifferentiated ES cells. Several observations, however, indicated that the absence of Dll4 was temporally affecting the emergence of mesodermal cells, an event known to occur at days 2–2.5 of EB differentiation [23]. First, Dll4^−/−^ EBs derived from independent Dll4 null ES clones persisted in an immature state for a longer period than their heterozygous and normal counterparts, as assessed by maintenance of an epiblast-like morphology at day 3, sustained expression of SSEA-1 until day 6, persistence of the primitive ectoderm marker *FGF5* until day 5, together with a delayed expression of *Gata6*, a primitive endoderm marker. Second, the expression of *Brachyury*, a transcription factor that marks the onset of mesoderm [28], was 24 hours delayed when compared with WT controls, a delay also occurring in the co-expression of SSEA-1 and Flk1, which is representative of early mesodermal progenitors [25]. These observations are consistent with recent reports in which undifferentiated murine ES cells were found to undergo mesoderm commitment when stimulated by exogenous Dll4 [43] and, also, that Delta-mediated signalling is required for mesoderm development in the sea urchin embryo [44]. In the latter study, expression of *Gcm*, a transcription factor required for mesoderm specification, was dependent on Delta-signalling only for a short period of time being thereafter independent of Delta activation. Our observation that Dll4 appears to be transiently up-regulated prior to mesoderm formation in normal EBs is reminiscent of the above findings, reinforcing the idea that Dll4 signalling not only regulates the timing of germ layer emergence but also promotes mesoderm formation during EB development. Also, the increased expression of *Pax6*, a marker of neuroectodermal progenitors, between days 5 and 8 in Dll4^+/−^ and Dll4^−/−^ EBs, further suggests that Dll4 also negatively regulates the formation of neuroectoderm in this system.

In agreement with the delay in *Brachyury* expression, a marked decrease in Flk1^+^ cells at days 3–3.5 was observed in Dll4^−/−^ EBs showing that the absence of Dll4 causes the almost complete disappearance of the early Flk1^+^ subpopulation where hemangioblasts are contained [38]. Accordingly, Dll4^−/−^ EBs generated reduced numbers of blast colonies, a phenotype that was partly rescued by the re-introduction of the Dll4 cDNA in the parental Dll4^−/−^ ES cells. As expected, a partial rescue of the early Flk1^+^ subpopulation was also observed. As all these observations would predict, Dll4^−/−^ EBs showed profound decrease in primitive and definitive hematopoietic progenitors. In contrast, in Dll4^+/−^ EBs, the kinetics and the frequency of Flk1^+^ cells were similar to the control at these time points, and the BL-CFC potential was also not significantly different from that of WT EBs. However, despite their nearly normal BL-CFC numbers, a severe reduction of EryP progenitors was found in Dll4^+/−^ EBs, while definitive hematopoiesis was not quantitatively or qualitatively affected. Analysis of the CFU potential of the YS from Dll4^+/−^ and Dll4^−/−^ embryos, further revealed that, *in vivo*, Dll4 hemizygosity also specifically affects primitive erythropoiesis. Therefore, the present data clearly indicate that two normal Dll4 alleles are essential for the quantitative regulation of embryonic EryP cells both *in vitro* and *in vivo*. These observations, together with previous studies in which the exposure of human fetal liver [45] or cord-blood [46] CD34^+^ cells to exogenous Dll4 was shown to promote the growth and differentiation of erythroid, but not of myeloid, committed progenitors, point to a special sensitivity of the erythroid lineage to Dll4-mediated signaling. Although the mechanistic basis for this phenomenon remains to be determined, we observed a significant reduction in the expression of *ephrin-B2* in Dll4^+/−^ EBs at day 4 of EB development (see Figure S4B). *Ephrin-B2* gene expression is regulated by Dll4-signaling [47], [48] and, ephrinB2 and its receptor EphB4, apart from their key role on arterial-venous specification in early embryogenesis [49], [50], are also known to positively influence the differentiation of erythropoietic progenitors, while not affecting that of non-erythroid precursors [51], [52]. Thus, it is possible that decreased levels of *EphrinB2* may contribute, at least in part, for the specific reduction of primitive erythroid colonies observed in Dll4^+/−^ EBs.

Of note, a primitive erythropoietic phenotype opposite to that described here has been observed by Hadland et al [9] in Notch1 null EBs, or following complete pharmacological abrogation of Notch-signaling at day 3.5 of EB development. While studies based on loss of function of Notch-receptors, or of Notch-ligands, cannot inform on which Notch-ligands or Notch-receptors, respectively, might be responsible for the observed effects, our findings, together with those of Hadland et al [9], are highly suggestive that the regulatory role of Notch-signaling on primitive erythropoiesis might be the end net result of differential actions exerted by distinct Notch-ligands. Specifically, the positive effect of Dll4 on the emergence of primitive erythropoietic progenitors might be counteracted by that of other ligands, akin to what has been shown for the opposing effects of Dll4 and Jagged1 on sprouting angiogenesis in the mouse neonatal retina [53]. Indeed, we did observe that three other Notch-ligands genes (Dll1, Jagged1 and, to a lesser extent, Jagged2) are strongly expressed in WT-EBs during the first 3 days of EB development (see Figure S4C). Our data obtained from OP9 co-cultures showed that feeder-layer over expression of Dll1 does not influence primitive erythropoiesis, either positively or negatively. These data are compatible with the hypothesis of a putative opposing effect of Jagged ligands with respect to the positive effect Dll4 has on primitive erythropoiesis.

Defects in endothelial remodeling are responsible for the embryonic lethality of mice harboring Dll4 heterozygous or homozygous deletions. The endothelial core of Dll4^+/−^ and Dll4^−/−^ blast colonies was grossly undistinguishable from that of control colonies, indicating that the specification of endothelial cells from the hemangioblast was not affected. However, as recently observed [54], when whole EBs develop endothelial sprouts through prolonged culture in methylcellulose medium, both Dll4^+/−^ and Dll4^−/−^ EBs show severe defects in vascular remodeling. Thus, similar to what is observed *in vivo* [15]–[17], heterozygous or homozygous deletions of Dll4 do not prevent the specification of the endothelial cells in developing EBs but severely affect their subsequent remodeling into normal vascular structures. In addition, a marked decrease in endothelial cells was observed in Dll4 null EBs from days 4.5 to 6, suggesting that Dll4 positively regulates the generation of endothelium from mesodermal endothelial progenitors [38].

Blast colonies have also been shown to give rise to vascular SMCs [40] and, a putative defect in the generation of this cell lineage could contribute to the abnormal angiogenic phenotype observed in Dll4 mutant EBs. We observed that Dll4^+/−^ and Dll4^−/−^ blast colonies cultured in conditions known to favor SMC differentiation originated SMCs at frequencies similar to those of control colonies, indicating that Dll4 is not necessary for the specification of this cell lineage from the hemangioblast. Additionally, cells isolated from day 3 Dll4^−/−^ EBs (in which Flk1^+^ cells are severely reduced) originated percentages of α-SMA^+^ cells similar to those of Dll4^+/−^ and WT EBs upon culture in matrigel-coated wells, further suggesting that SMC progenitors are present at normal numbers within the Flk1^+^ cells that appear in Dll4^−/−^ EBs at days 4–4.5. Together, these observations agree with previous reports showing that the differentiation of SMCs from murine embryonic fibroblasts (MEFs) is Dll4-independent [55] and, also, that Notch signaling is not required for the specification of SMCs from nascent mesoderm in chick embryos [56].

Given that the second Flk1^+^ population in developing EBs also has the potential to generate endothelial and cardiac cells [38], the emergence of these two cell lineages was monitored in Dll4 mutant EB cells. Expression analysis of Flk1 and Tie2, two endothelial cell markers that are sequentially expressed during EB development [57] revealed that endothelial cells in Dll4^−/−^ EBs were consistently reduced throughout EB differentiation, while in Dll4^+/−^ EBs a reduction was only visible at day 5, when compared to WT EBs. On the other hand, we observed that the proportion of EBs exhibiting contractile regions was increased in both Dll4^+/−^ and Dll4^−/−^ EBs. Previous studies have shown that Notch activity negatively regulates the differentiation of ES cells into cardiomyocytes [58], [59]. The present data further indicates that such an action might be mediated, at least in part, by Dll4, an assumption that is consistent with the observation that the Dll4 gene is highly expressed from days 4 to 8 in developing WT-EBs, when compared to other Notch-ligand genes (see Figure 1A and Figure S4C).

In summary, the present observations assign a role for the Notch ligand Dll4 at early stages of hemato-vascular development, namely in the generation of hemangioblasts and EryP progenitors. They further show that Dll4 participates, in a dose dependent manner, in the temporal regulation of mesoderm formation and, subsequently, differentially regulates other mesoderm-derived cell lineages, favoring endothelial cell differentiation and inhibiting cardiomyogenesis while not affecting SMC specification. Further studies will be required to identify the mechanisms by which Dll4 exerts these functions and how they relate with other major signaling pathways crucial for early embryogenesis and hemato-vascular development.

## Materials and Methods

### Ethics Statement

All animal-involving procedures in this study were approved by the Ethics and Animal Welfare Committee from the Lisbon Faculty of Veterinary Medicine (Permit number: CVT/71084/2006).

### ES cell lines and EB differentiation

Two previously described murine ES cell lines, R1 [60] and its Dll4^+/−^ derivative [15], were used in this study. To obtain Dll4^−/−^ ES cells, the Dll4^+/−^ cell line was cultured on G418-resistant MEFs (DR4, ATCC) in the presence of 1.5 mg/ml of G418 (Sigma-Aldrich). After 15 days of culture, G418-resistant ES cell clones were selected and expanded. To confirm the loss of the wild-type (WT) Dll4 allele, PCR genotyping was performed as described [15]. Three Dll4^−/−^ ES cell clones (#7, #9i and #10i) were selected and used for further studies.

Parental R1 (WT), Dll4^+/−^ and Dll4^−/−^ ES cell lines were maintained on mitomycin C-inactivated MEFs in Dulbecco's Modified Eagle's Medium (DMEM, Sigma-Aldrich) supplemented with 15% fetal bovine serum (FBS), 0.1 mM β-mercaptoethanol (GIBCO-Invitrogen), 1000 U/mL recombinant leukemia inhibitory factor (Chemicon), 1% L-glutamine, 1% penicillin-streptomycin, 1% non-essential amino acids and 1% nucleosides (Sigma-Aldrich).

Two days prior the onset of differentiation, ES cells were transferred to Iscove's Modified Dulbecco's Medium (IMDM, Sigma-Aldrich) containing the above components. EBs in suspension culture were then generated as described [61] and, for differentiation in a semi-solid medium, ES cells were plated in the same medium supplemented with 1% methylcellulose (M3134, StemCell Technologies) and 40 ng/ml SCF (Peprotech). Differentiation of ES cells on OP9 stromal cells was performed as described [37].

### Mouse embryos

Embryos were obtained by crossing CD1 (WT) mice with Dll4^+/−^ mice in the CD1 genetic background or by the intercross of Dll4^+/−^ mice as previously described [15]. The YS was dissected from the embryo proper, which was used for PCR genotyping [15]. Embryonic age was determined by the number of somites.

### Colony assays

Generation of blast colonies was performed as described [2], except for the absence of D4T conditioned medium. For the growth of hematopoietic progenitors, EBs or YS were dissociated with 0.25% trypsin and the derived cells plated in 1% methylcellulose with cytokines (M3434, StemCell Technologies). Hematopoietic colony morphology was confirmed in May-Grünwald-Giemsa-stained cytospins.

### Endothelial and smooth muscle differentiation

Day 3 EB-derived cells and blast colonies with 4 days of development were cultured as described [39], except for the absence of D4T conditioned medium.

### Retroviral transduction of Dll4^−/−^ ES cells

The mDll4 cDNA was cloned into the EcoRI site of the enhanced green fluorescent protein (eGFP)-expressing retroviral vector pMigR1. Empty (pMigR1) or recombinant virus (pMigR1-Dll4) were obtained as described [62] followed by spin-infection [34] of Dll4^−/−^ ES cells (clone #7). After 48 hours of pre-differentiation [61], GFP^+^ cells were sorted on a FACSAria (Becton, Dickinson and Company (BD)) to >95% purity and differentiated into EBs.

### Flow cytometry

Cells were stained with the following mouse antibodies: anti-SSEA-1-Alexa Fluor 488, anti-Flk1-PE, anti-CD41-PE, anti-Tie2-biotin, anti-PdgfRα - APC, anti-VE-cadherin-APC (eBioscience). Biotin was detected with FITC-conjugated streptavidin (eBioscience). Sorting of Flk1^+^ cells was performed with FACSAria instrument (BD). Non-viable cells were excluded from the analysis by 7-AAD (eBioscience) staining. For proliferation analysis, cells were stained with PE Mouse Anti-human Ki-67 according to the manufacturer's instructions (BD Pharmingen). Apoptosis was assessed using Annexin V/propidium iodide (PI) detection kit (eBioscience). Cells were analyzed on a FACSCalibur (BD) and data analyzed using FlowJo software (TreeStar).

### Immunocytochemistry and image analysis

Detection of E-cadherin during EB development was done in three-dimensionally preserved EBs as previously described [63]. For SMCs identification, cells were fixed in 3.7% PFA/PBS, permeabilized in 0.2% Triton X-100/PBS, blocked in 3% BSA/0.1% Tween 20/PBS, incubated with anti-α-SMA antibody (Abcam) followed by FITC-conjugated secondary antibody. EBs in methylcellulose medium were fixed in 3.7% PFA/PBS, blocked in 3% BSA/PBS and incubated with anti-CD31-biotin antibody (BD Pharmingen) followed by FITC-conjugated streptavidin. All preparations were mounted in Vectashield-DAPI (Vector Laboratories) and visualized with a Leica DM5000B (Leica) epifluorescence microscope or with a confocal microscope Zeiss LSM510 META (Carl Zeiss).

### Semi-quantitative and quantitative RT-PCR

Total RNA was isolated with the High Pure RNA Isolation Kit (Roche Diagnostics), followed by reverse transcription of 2 µg of RNA with the SuperScript III First-Strand Synthesis System (Invitrogen) using oligo(dT) primers. For semi-quantitative PCR, conditions for each gene were optimized in order to detect the exponential phase of amplification. Glyceraldehyde 3 phosphate dehydrogenase (GAPDH) was used as a reference for the amount of cDNA in each lane. PCR products were resolved on 1.5% agarose gels and visualized by GelRed (Biotium) staining. Primers and PCR conditions are listed in Table S1. Quantitative RT-PCR (qRT-PCR) was performed with SYBR Green PCR Master Mix (Applied BioSystems) and primers (Brachyury- PPM05479E; Ephrin B2 - PPM03657B; GAPDH- PPM02946E; SA biosciences) on an ABI PRISM 7500 Fast Real Time PCR System (Applied BioSystems). The comparative Ct (2-ΔΔCt) method was used with GAPDH gene as an endogenous control and day 2 WT EB- derived sample as a calibrator. Three to five biological replicates were analyzed for each condition.

### Statistical analysis

All experiments were performed at least three times, and data are expressed as mean ± SD. Data were analyzed by unpaired Student's t-test or one-way ANOVA using GraphPad Prism 5.0 software (GraphPad Software). Values of P less than .05 were considered significant.

## Supporting Information

Figure S1 **Effects of Dll4 in ES cell maintenance and EB generation.** (A) Proliferation of WT, Dll4^+/−^ and Dll4^−/−^ ES cells cultured for 4 days on MEFs. (B) Number of EBs generated by WT, Dll4^+/−^ and Dll4^−/−^ ES cells when cultured in suspension. (C) Cellularity of WT, Dll4^+/−^ and Dll4^−/−^ EBs when cultured in suspension from day 2 to day 6. (D–E) Cell proliferation (D) and apoptosis (E) in WT, Dll4^+/−^ and Dll4^−/−^ EBs, from day 2 to day 6, as determined by the percentage of Ki-67^+^ cells and Annexin V^+^ cells, respectively. (F) Flow cytometry analysis of SSEA-1 expression in WT, Dll4^+/−^ and Dll4^−/−^ EBs from day 0 to day 6. ∇: 0.0000086<*P*<0.048246 from day 3.5 to day 6. (G) Quantitative RT-PCR analysis of *Brachyury* in WT, Dll4^+/−^ and Dll4^−/−^ EBs from day 2 to day 6 of differentiation. ▴: WT and Dll4^+/−^ *versus* all Dll4^−/−^, 0.0004<*P*<0.0090.
(TIF)

Figure S2 **Effects of Dll4 on the emergence of SSEA-1^+^ cells, Flk1^+^ cells, hematopoietic and endothelial cells.** (A–E) Flow cytometry analysis of SSEA-1^+^ (A), SSEA-1^+^/Flk1^+^ (B), Flk1^+^ (C) CD41^+^ (D) and Tie2^+^/Flk1^+^/CD41^−^ (E) cells in WT, Dll4^+/−^ and Dll4^−/−^ EBs. Absolute cell numbers counted at the indicated days. ND not determined. ▴: WT and Dll4^+/−^ *versus* #7, *P* = 0.005276 and 0.002622, respectively; □: WT and Dll4^+/−^ *versus* #7 and #10i, 0.013904<*P*<0.046047. ∇: day 3 – WT and Dll4^+/−^ *versus* #7 and #9i, 0.0376<*P*<0.0459; day 3.5 – WT *versus* all Dll4^−/−^, 0.0228<*P*<0.0286; ◊: day 3 – WT and Dll4^+/−^ *versus* all Dll4^−/−^ cells, 0.01206<*P*<0.0248.**▾**: day 4 – WT *versus* all Dll4^−/−^ cells, 0.02517<*P*<0.03789; day 4.5 – WT and Dll4^+/−^ *versus* all Dll4^−/−^ cells, 0.003056<*P*<0.042568; day 5 – Dll4^+/−^ and #7 *versus* WT, *P* = 0.012558 and 0.00922, respectively; day 6 – WT and Dll4^+/−^ *versus* all Dll4^−/−^, 0.001036<*P*<0.04778. ▪: day 5 – Dll4^+/−^ *versus* #7, *P* = 0.02404; day 6 – WT *versus* #7 and #10i, *P* = 0.03235 and 0.04762, respectively.
(TIF)

Figure S3 **Blast potential of Dll4^−/−^ cells.** (A) Number of blast colonies generated by day 3 WT and Dll4^−/−^ EB-cells. Similar numbers of EBs were generated by three independent Dll4^−/−^ clones (#7, #9i, #10i). (B) Flow cytometry analysis of Flk1^+^ cells derived from day 3 EBs differentiated from Dll4**^−^** ^/**−**^ ES cells transduced with empty (pMigR1) or recombinant virus (pMigR1-Dll4). *P<.05, ***P<.001.
(TIF)

Figure S4 **Gene expression analysis in WT, Dll4^+/−^ and Dll4^−/−^ differentiating EBs.** (A) Semi-quantitative RT-PCR analysis of *Scl*, *Gata1* and *Runx1* in WT, Dll4^+/−^ and Dll4^−/−^ EBs from day 0 to day 8 of differentiation. (B) Quantitative RT-PCR analysis of *Ephrin B2* in WT, Dll4^+/−^ and Dll4^−/−^ EBs from day 2 to day 6 of differentiation. ND not determined. *P<.05, **P<.01. (C) Semi-quantitative RT-PCR analysis of Notch-ligand genes *Dll1*, *Jagged1* and *Jagged2* in WT EBs from day 0 to day 8 of differentiation.
(TIF)

Table S1 **Primer sequences and conditions used for semi-quantitative RT-PCR detection of gene expression of relevant Notch-system components, transcription factors and germ-layer markers, used in this study.**
(DOC)

## References

[1] Palis J, Yoder MC (2001). Yolk-sac hematopoiesis: the first blood cells of mouse and man.. Exp Hematol.

[2] Kennedy M, Firpo M, Choi K, Wall C, Robertson S (1997). A common precursor for primitive erythropoiesis and definitive haematopoiesis.. Nature.

[3] Choi K, Kennedy M, Kazarov A, Papadimitriou JC, Keller G (1998). A common precursor for hematopoietic and endothelial cells.. Development.

[4] Huber TL, Kouskoff V, Fehling HJ, Palis J, Keller G (2004). Haemangioblast commitment is initiated in the primitive streak of the mouse embryo.. Nature.

[5] Cumano A, Godin I (2007). Ontogeny of the hematopoietic system.. Annu Rev Immunol.

[6] Radtke F, Wilson A, Mancini SJ, MacDonald HR (2004). Notch regulation of lymphocyte development and function.. Nat Immunol.

[7] Ohishi K, Katayama N, Shiku H, Varnum-Finney B, Bernstein ID (2003). Notch signalling in hematopoiesis.. Semin Cell Dev Biol.

[8] Kumano K, Chiba S, Kunisato A, Sata M, Saito T (2003). Notch1 but not Notch2 is essential for generating hematopoietic stem cells from endothelial cells.. Immunity.

[9] Hadland BK, Huppert SS, Kanungo J, Xue Y, Jiang R (2004). A requirement for Notch1 distinguishes 2 phases of definitive hematopoiesis during development.. Blood.

[10] Schroeder T, Meier-Stiegen F, Schwanbeck R, Eilken H, Nishikawa S (2006). Activated Notch1 alters differentiation of embryonic stem cells into mesodermal cell lineages at multiple stages of development.. Mech Dev.

[11] Lee D, Park C, Lee H, Lugus JJ, Kim SH (2008). ER71 acts downstream of BMP, Notch, and Wnt signaling in blood and vessel progenitor specification.. Cell Stem Cell.

[12] Cheng X, Huber TL, Chen VC, Gadue P, Keller GM (2008). Numb mediates the interaction between Wnt and Notch to modulate primitive erythropoietic specification from the hemangioblast.. Development.

[13] Chen VC, Stull R, Joo D, Cheng X, Keller G (2008). Notch signaling respecifies the hemangioblast to a cardiac fate.. Nat Biotechnol.

[14] Del Monte G, Grego-Bessa J, González-Rajal A, Bolós V, De La Pompa JL (2007). Monitoring Notch1 activity in development: evidence for a feedback regulatory loop.. Dev Dyn.

[15] Duarte A, Hirashima M, Benedito R, Trindade A, Diniz P (2004). Dosage-sensitive requirement for mouse Dll4 in artery development.. Genes Dev.

[16] Krebs LT, Shutter JR, Tanigaki K, Honjo T, Stark KL (2004). Haploinsufficient lethality and formation of arteriovenous malformations in Notch pathway mutants.. Genes Dev.

[17] Gale NW, Dominguez MG, Noguera I, Pan L, Hughes V (2004). Haploinsufficiency of delta-like 4 ligand results in embryonic lethality due to major defects in arterial and vascular development.. Proc Natl Acad Sci U S A.

[18] Jaffredo T, Nottingham W, Liddiard K, Bollerot K, Pouget C (2005). From hemangioblast to hematopoietic stem cell: an endothelial connection?. Exp Hematol.

[19] Eilken HM, Nishikawa S, Schroeder T (2009). Continuous single-cell imaging of blood generation from haemogenic endothelium.. Nature.

[20] Lancrin C, Sroczynska P, Stephenson C, Allen T, Kouskoff V (2009). The haemangioblast generates haematopoietic cells through a haemogenic endothelium stage.. Nature.

[21] Keller G, Kennedy M, Papayannopoulou T, Wiles MV (1993). Hematopoietic commitment during embryonic stem cell differentiation in culture.. Mol Cell Biol.

[22] Keller G (2005). Embryonic stem cell differentiation: emergence of a new era in biology and medicine.. Genes Dev.

[23] Fehling HJ, Lacaud G, Kubo A, Kennedy M, Robertson S (2003). Tracking mesoderm induction and its specification to the hemangioblast during embryonic stem cell differentiation.. Development.

[24] Solter D, Knowles BB (1978). Monoclonal antibody defining a stage-specific mouse embryonic antigen (SSEA-1).. Proc Natl Acad Sci USA.

[25] Yue W, Pi QM, Zhang WJ, Zhou GD, Cui L (2010). Platelet endothelial cell adhesion molecule-1, stage-specific embryonic antigen-1, and Flk-1 mark distinct populations of mouse embryonic stem cells during differentiation toward hematopoietic/endothelial cells.. Stem Cells Dev.

[26] Rogers MB, Hosler BA, Gudas LJ (1991). Specific expression of a retinoic acid-regulated, zinc-finger gene, Rex-1, in preimplantation embryos, trophoblast and spermatocytes.. Development.

[27] Haub O, Goldfarb M (1991). Expression of the fibroblast growth factor-5 gene in the mouse embryo.. Development.

[28] Wilkinson DG, Bhatt S, Herrmann BG (1990). Expression pattern of the mouse T gene and its role in mesoderm formation.. Nature.

[29] Morrisey EE, Tang Z, Sigrist K, Lu MM, Jiang F (1998). GATA6 regulates HNF4 and is required for differentiation of visceral endoderm in the mouse embryo.. Genes Dev.

[30] Li H, Liu H, Corrales CE, Risner JR, Forrester J (2009). Differentiation of neurons from neural precursors generated in floating spheres from embryonic stem cells.. BMC Neurosci.

[31] Kabrun N, Bühring HJ, Choi K, Ullrich A, Risau W (1997). Flk-1 expression defines a population of early embryonic hematopoietic precursors.. Development.

[32] Ishitobi H, Wakamatsu A, Liu F, Azami T, Hamada M (2011). Molecular basis for Flk1 expression in hemato-cardiovascular progenitors in the mouse.. Development.

[33] D'Souza SL, Elefanty AG, Keller G (2005). SCL/Tal-1 is essential for hematopoietic commitment of the hemangioblast but not for its development.. Blood.

[34] Cherry SR, Biniszkiewicz D, van Parijs L, Baltimore D, Jaenisch R (2000). Retroviral expression in embryonic stem cells and hematopoietic stem cells.. Mol Cell Biol.

[35] Kattman SJ, Witty AD, Gagliardi M, Dubois NC, Niapour M (2011). Stage-specific optimization of activin/nodal and BMP signaling promotes cardiac differentiation of mouse and human pluripotent stem cell lines.. Cell Stem Cell.

[36] Ferkowicz MJ, Starr M, Xie X, Li W, Johnson SA (2003). CD41 expression defines the onset of primitive and definitive hematopoiesis in the murine embryo.. Development.

[37] Nakano T, Kodama H, Honjo T (1994). Generation of lymphohematopoietic cells from embryonic stem cells in culture.. Science.

[38] Kattman SJ, Huber TL, Keller GM (2006). Multipotent flk-1+ cardiovascular progenitor cells give rise to the cardiomyocyte, endothelial, and vascular smooth muscle lineages.. Dev Cell.

[39] Kubo A, Chen V, Kennedy M, Zahradka E, Daley GQ (2005). The homeobox gene HEX regulates proliferation and differentiation of hemangioblasts and endothelial cells during ES cell differentiation.. Blood.

[40] Ema M, Faloon P, Zhang WJ, Hirashima M, Reid T (2003). Combinatorial effects of Flk1 and Tal1 on vascular and hematopoietic development in the mouse.. Genes Dev.

[41] Kouskoff V, Lacaud G, Schwantz S, Fehling HJ, Keller G (2005). Sequential development of hematopoietic and cardiac mesoderm during embryonic stem cell differentiation.. Proc Natl Acad Sci U S A.

[42] Tanaka M, Chen Z, Bartunkova S, Yamasaki N, Izumo S (1999). The cardiac homeobox gene Csx/Nkx2.5 lies genetically upstream of multiple genes essential for heart development.. Development.

[43] Ramasamy SK, Lenka N (2010). Notch exhibits ligand bias and maneuvers stage-specific steering of neural differentiation in embryonic stem cells.. Mol Cell Biol.

[44] Croce JC, McClay DR (2010). Dynamics of Delta/Notch signaling on endomesoderm segregation in the sea urchin embryo.. Development.

[45] Dando JS, Tavian M, Catelain C, Poirault S, Bennaceur-Griscelli A (2005). Notch/Delta4 interaction in human embryonic liver CD34+ CD38- cells: positive influence on BFU-E production and LTC-IC potential maintenance.. Stem Cells.

[46] Sugimoto A, Yamamoto M, Suzuki M, Inoue T, Nakamura S (2006). Delta-4 Notch ligand promotes erythroid differentiation of human umbilical cord blood CD34+ cells.. Exp Hematol.

[47] Iso T, Maeno T, Oike Y, Yamazaki M, Doi H (2006). Dll4-selective Notch signaling induces ephrinB2 gene expression in endothelial cells..

[48] Masumura T, Yamamoto K, Shimizu N, Obi S, Ando J (2009). Shear stress increases expression of the arterial endothelial marker ephrinB2 in murine ES cells via the VEGF-Notch signaling pathways.. Arterioscler Thromb Vasc Biol.

[49] Wang HU, Chen ZF, Anderson DJ (1998). Molecular distinction and angiogenic interaction between embryonic arteries and veins revealed by ephrin-B2 and its receptor Eph-B4.. Cell.

[50] Pitulescu ME, Adams RH (2010). Eph/ephrin molecules - a hub for signaling and endocytosis.. Genes Dev.

[51] Wang Z, Miura N, Bonelli A, Mole P, Carlesso N (2001). Receptor tyrosine kinase, EphB4 (HTK), accelerates differentiation of select human hematopoietic cells.. Blood.

[52] Suenobu S, Takakura N, Inada T, Yamada Y, Yuasa H (2002). A role of EphB4 receptor and its ligand, ephrin-B2, in erythropoiesis.. Biochemical and Biophysical Research Communications.

[53] Benedito R, Roca C, Sörensen I, Adams S, Gossler A (2009). The Notch ligands Dll4 and Jagged1 have opposing effects on angiogenesis.. Cell.

[54] Jakobsson L, Franco CA, Bentley K, Collins RT, Ponsioen B (2010). Endothelial cells dynamically compete for the tip cell position during angiogenic sprouting.. Nat Cell Biol.

[55] Doi H, Iso T, Sato H, Yamazaki M, Matsui H (2006). Jagged1-selective notch signaling induces smooth muscle differentiation via a RBP-Jkappa-dependent pathway.. J Biol Chem.

[56] Shin M, Nagai H, Sheng G (2009). Notch mediates Wnt and BMP signals in the early separation of smooth muscle progenitors and blood/endothelial common progenitors.. Development.

[57] Vittet D, Prandini MH, Berthier R, Schweitzer A, Martin-Sisteron H (1996). Embryonic stem cells differentiate in vitro to endothelial cells through successive maturation steps.. Blood.

[58] Schroeder T, Fraser ST, Ogawa M, Nishikawa S, Oka C (2003). Recombination signal sequence-binding protein Jkappa alters mesodermal cell fate decisions by suppressing cardiomyogenesis.. Proc Natl Acad Sci U S A.

[59] Nemir M, Croquelois A, Pedrazzini T, Radtke F (2006). Induction of cardiogenesis in embryonic stem cells via downregulation of Notch1 signaling.. Circ Res.

[60] Nagy A, Rossant J, Nagy R, Abramow-Newerly W, Roder JC (1993). Derivation of completely cell culture-derived mice from early-passage embryonic stem cells.. Proc Natl Acad Sci USA.

[61] Lacaud G, Gore L, Kennedy M, Kouskoff V, Kingsley P (2002). Runx1 is essential for hematopoietic commitment at the hemangioblast stage of development in vitro.. Blood.

[62] Jaleco AC, Neves H, Hooijberg E, Gameiro P, Clode N (2001). Differential effects of Notch ligands Delta-1 and Jagged-1 in human lymphoid differentiation.. J Exp Med.

[63] Alcobia I, Gomes A, Saavedra P, Laranjeiro R, Oliveira S (2010). Portrayal of the Notch system in embryonic stem cell-derived embryoid bodies.. Cells Tissues Organs.

