# Supplementary figures and images for: The Notch Ligand Delta-Like 4 Regulates Multiple Stages of Early Hemato-Vascular Development
Source: PLoS One. 2012 Apr 13;7(4):e34553. doi: 10.1371/journal.pone.0034553 (PMC3326024; doi:10.1371/journal.pone.0034553)

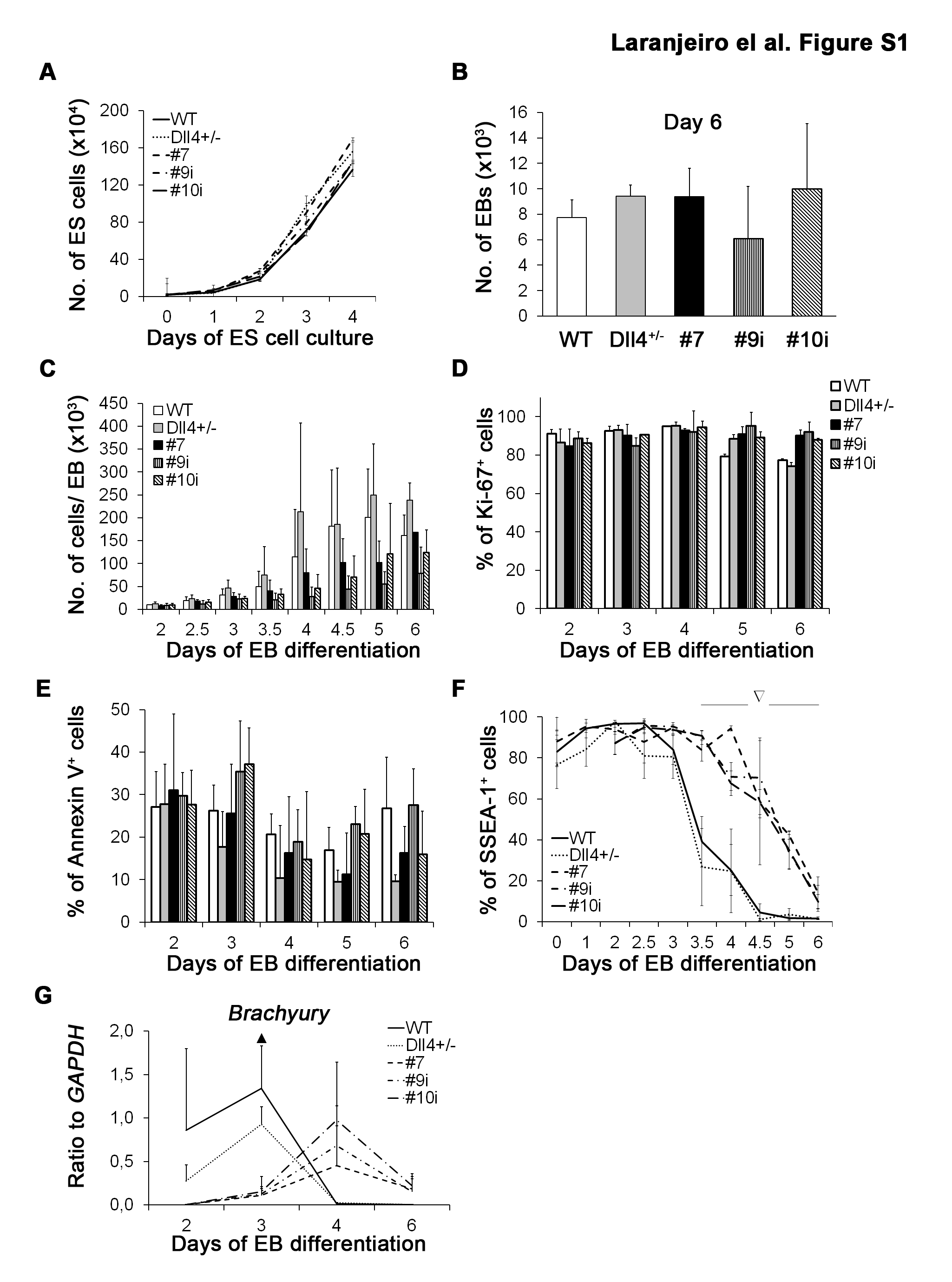

Supplement: Figure S1 — Effects of Dll4 in ES cell maintenance and EB generation. (A) Proliferation of WT, Dll4+/− and Dll4−/− ES cells cultured for 4 days on MEFs. (B) Number of EBs generated by WT, Dll4+/− and Dll4−/− ES cells when cultured in suspension. (C) Cellularity of WT, Dll4+/− and Dll4−/− EBs when cultured in suspension from day 2 to day 6. (D–E) Cell proliferation (D) and apoptosis (E) in WT, Dll4+/− and Dll4−/− EBs, from day 2 to day 6, as determined by the percentage of Ki-67+ cells and Annexin V+ cells, respectively. (F) Flow cytometry analysis of SSEA-1 expression in WT, Dll4+/− and Dll4−/− EBs from day 0 to day 6. ∇: 0.0000086<P<0.048246 from day 3.5 to day 6. (G) Quantitative RT-PCR analysis of Brachyury in WT, Dll4+/− and Dll4−/− EBs from day 2 to day 6 of differentiation. ▴: WT and Dll4+/− versus all Dll4−/−, 0.0004<P<0.0090. (TIF) [file pone.0034553.s001.tif]

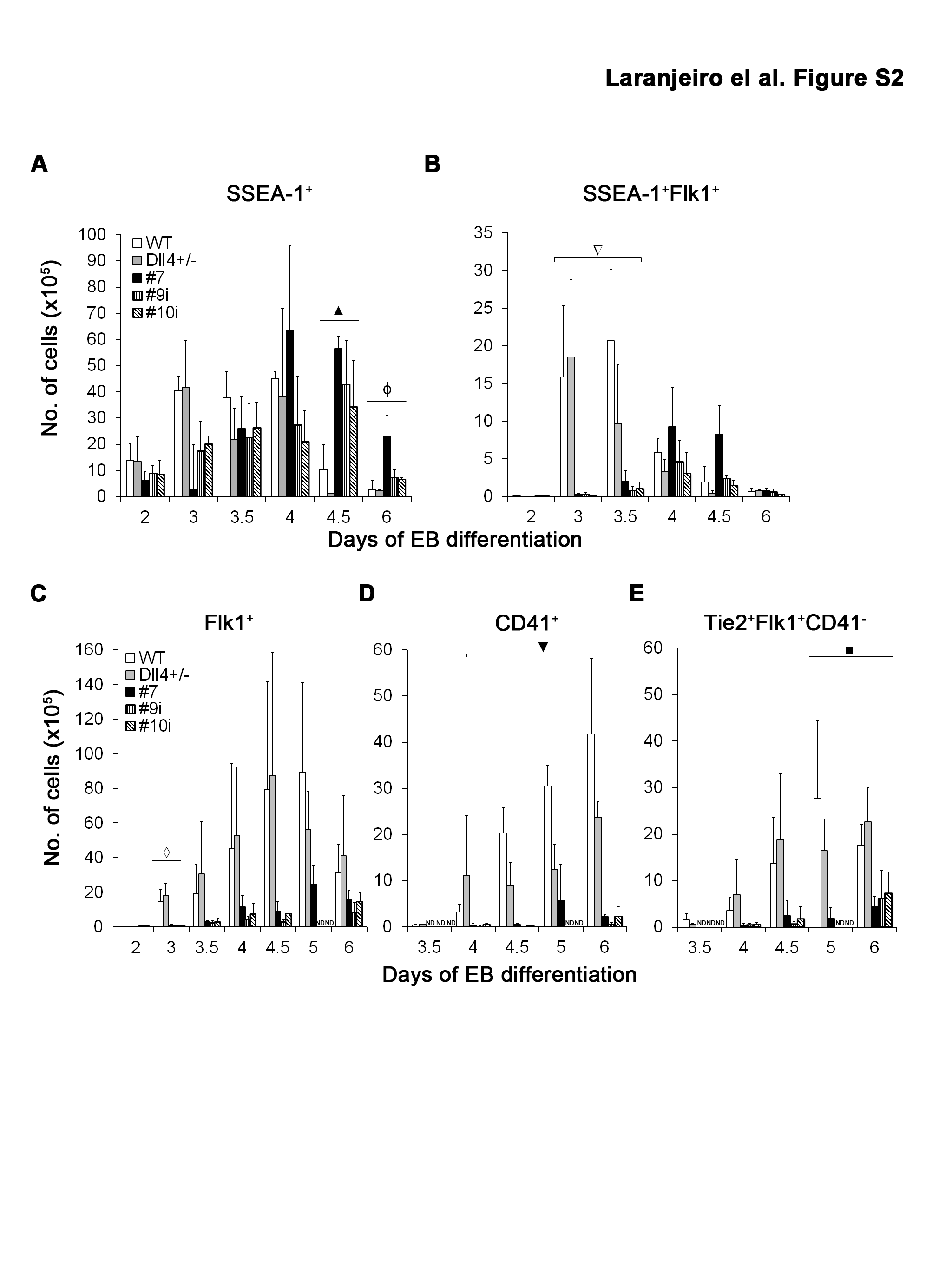

Supplement: Figure S2 — Effects of Dll4 on the emergence of SSEA-1+ cells, Flk1+ cells, hematopoietic and endothelial cells. (A–E) Flow cytometry analysis of SSEA-1+ (A), SSEA-1+/Flk1+ (B), Flk1+ (C) CD41+ (D) and Tie2+/Flk1+/CD41− (E) cells in WT, Dll4+/− and Dll4−/− EBs. Absolute cell numbers counted at the indicated days. ND not determined. ▴: WT and Dll4+/− versus #7, P = 0.005276 and 0.002622, respectively; □: WT and Dll4+/− versus #7 and #10i, 0.013904<P<0.046047. ∇: day 3 – WT and Dll4+/− versus #7 and #9i, 0.0376<P<0.0459; day 3.5 – WT versus all Dll4−/−, 0.0228<P<0.0286; ◊: day 3 – WT and Dll4+/− versus all Dll4−/− cells, 0.01206<P<0.0248.▾: day 4 – WT versus all Dll4−/− cells, 0.02517<P<0.03789; day 4.5 – WT and Dll4+/− versus all Dll4−/− cells, 0.003056<P<0.042568; day 5 – Dll4+/− and #7 versus WT, P = 0.012558 and 0.00922, respectively; day 6 – WT and Dll4+/− versus all Dll4−/−, 0.001036<P<0.04778. ▪: day 5 – Dll4+/− versus #7, P = 0.02404; day 6 – WT versus #7 and #10i, P = 0.03235 and 0.04762, respectively. (TIF) [file pone.0034553.s002.tif]

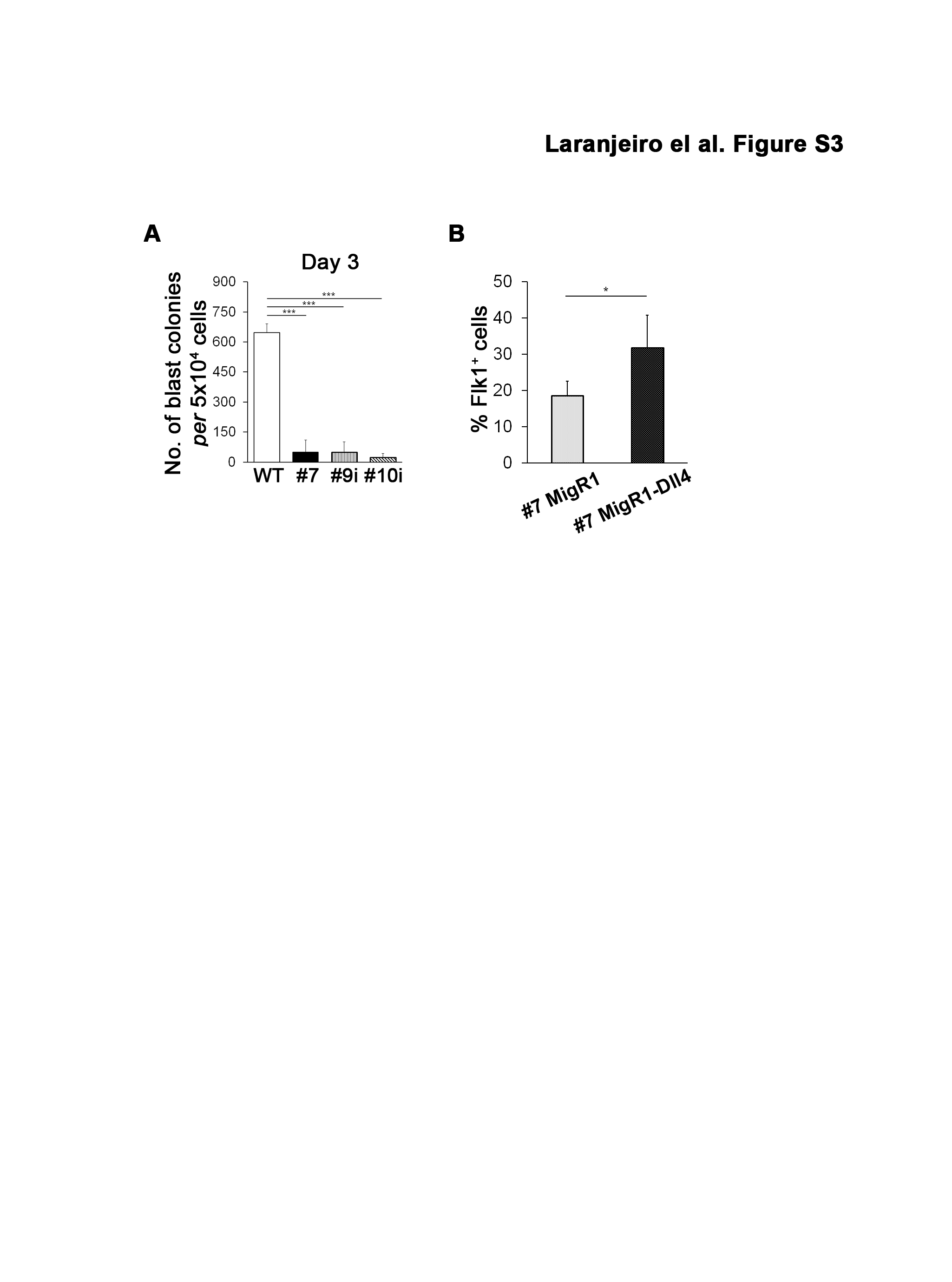

Supplement: Figure S3 — Blast potential of Dll4−/− cells. (A) Number of blast colonies generated by day 3 WT and Dll4−/− EB-cells. Similar numbers of EBs were generated by three independent Dll4−/− clones (#7, #9i, #10i). (B) Flow cytometry analysis of Flk1+ cells derived from day 3 EBs differentiated from Dll4− /− ES cells transduced with empty (pMigR1) or recombinant virus (pMigR1-Dll4). *P<.05, ***P<.001. (TIF) [file pone.0034553.s003.tif]

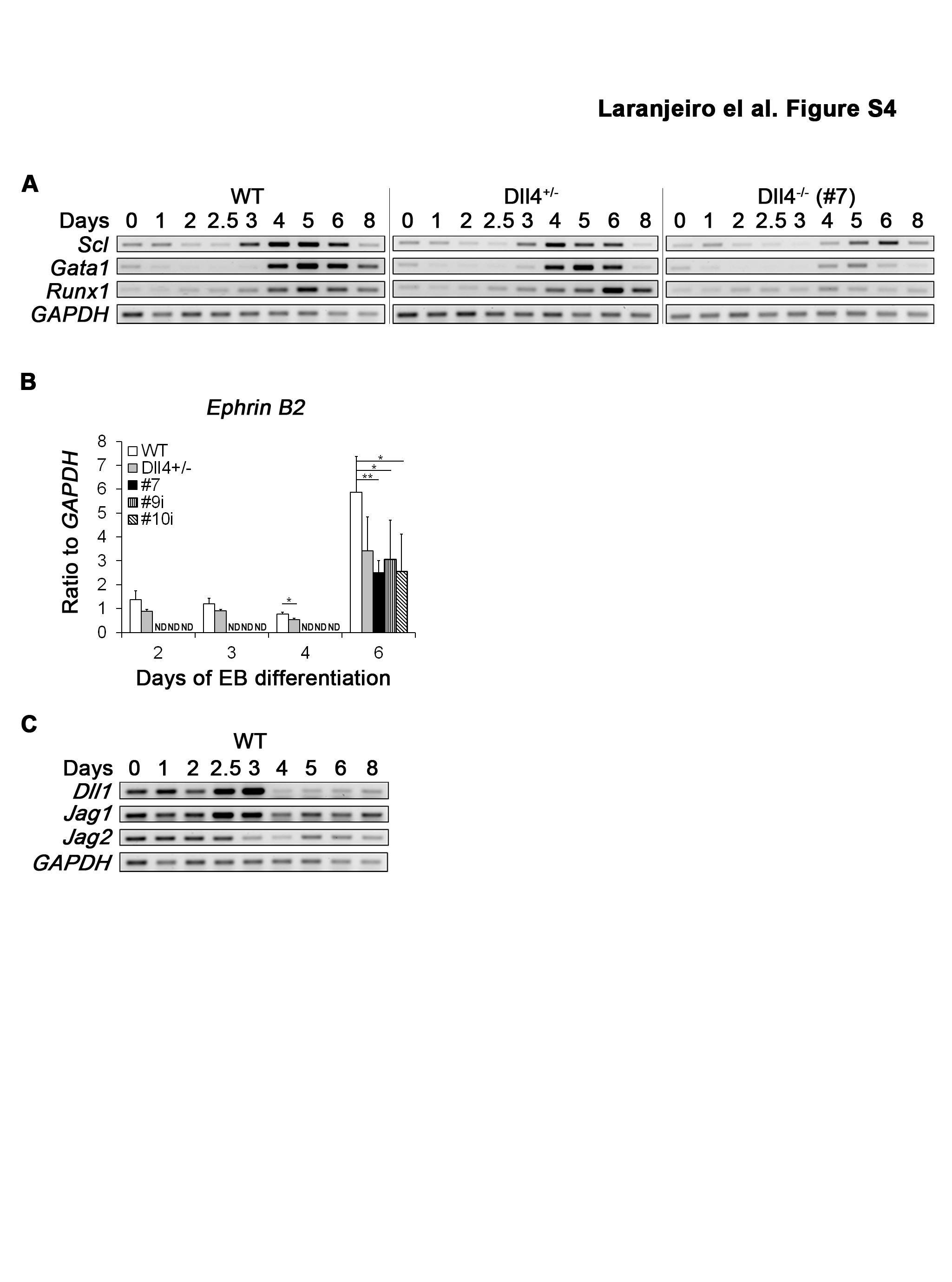

Supplement: Figure S4 — Gene expression analysis in WT, Dll4+/− and Dll4−/− differentiating EBs. (A) Semi-quantitative RT-PCR analysis of Scl, Gata1 and Runx1 in WT, Dll4+/− and Dll4−/− EBs from day 0 to day 8 of differentiation. (B) Quantitative RT-PCR analysis of Ephrin B2 in WT, Dll4+/− and Dll4−/− EBs from day 2 to day 6 of differentiation. ND not determined. *P<.05, **P<.01. (C) Semi-quantitative RT-PCR analysis of Notch-ligand genes Dll1, Jagged1 and Jagged2 in WT EBs from day 0 to day 8 of differentiation. (TIF) [file pone.0034553.s004.tif]
