# Supplementary material for: The Notch Ligand Delta-Like 4 Regulates Multiple Stages of Early Hemato-Vascular Development
Source: PLoS One. 2012 Apr 13;7(4):e34553. doi: 10.1371/journal.pone.0034553 (PMC3326024; doi:10.1371/journal.pone.0034553)
Supplement: Table S1 — Primer sequences and conditions used for semi-quantitative RT-PCR detection of gene expression of relevant Notch-system components, transcription factors and germ-layer markers, used in this study. (DOC) [file pone.0034553.s005.doc]

**Table S1. Primers and conditions used for semi-quantitative RT-PCR.**

| **Gene** | **Forward primer (5'-3')** | **Reverse primer (5'-3')** | **Product**  **size (bp)** | **Annealing**  **temp (ºC)** | **Cycles** |
| --- | --- | --- | --- | --- | --- |
| ***Brachyury*** | CATGTACTCTTTCTTGCTGG | GGTCTCGGGAAAGCAGTGGC | 313 | 62 | 28 |
| ***Dll1*** | GGCCTTTTCTGCAACCAAGAC | GGAACATGTGTAGCTCCCCTG | 102 | 60 | 31 |
| ***Dll4*** | ACCTTTGGCAATGTCTCCAC | GTTTCCTGGCGAAGTCTCTG | 189 | 60 | 36 |
| ***FGF5*** | ACCCGGATGGCAAAGTCAA | CAATCCCCTGAGACACAGCAA | 81 | 60 | 29 |
| ***GAPDH*** | CATTGTGGAAGGGCTCATGA | GCCCCACGGCCATCA | 99 | 60 | 25 |
| ***Gata1*** | GAAGCGAATGATTGTCAGCA | TTCCTCGTCTGGATTCCATC | 183 | 60 | 33 |
| ***Gata6*** | GACGGCACCGGTCATTACC | ACAGTTGGCACAGGACAGTCC | 132 | 60 | 30 |
| ***Jag1*** | CACGTGGCCATCTCTGCAG | ACCGCAGCAATAAGTGAGCTGT | 119 | 60 | 30 |
| ***Jag2*** | AGGTGCCAGGAAGTGGTCATA | CATCCGCACCATACCTTGC | 143 | 60 | 30 |
| ***Nkx2-5*** | AAAGAGCTGTGCGCGCT | GCTGTCGCTTGCACTTGTAG | 259 | 58 | 32 |
| ***Pax6*** | GAGAAGAGAAGAGAAACTGAGGAACCAGA | ATGGGTGGCAAAGCACTGTACG | 201 | 60 | 35 |
| ***Rex1*** | CCTGCACACAGAAGAAAGCA | CCACTTGTCTTTGCCGTTTT | 203 | 60 | 31 |
| ***Runx1*** | CTCGGCAGAACTGAGAAATG | GACGGTGATGGTCAGAGTGA | 118 | 60 | 32 |
| ***SCL*** | AACAACAACCGGGTGAAGAG | AGGCGGAGGATCTCATTCTT | 206 | 60 | 33 |
